# Measuring Faecal Epi-Androsterone as an Indicator of Gonadal Activity in Spotted Hyenas (*Crocuta crocuta*)

**DOI:** 10.1371/journal.pone.0128706

**Published:** 2015-06-24

**Authors:** Susanne Pribbenow, Marion L. East, Andre Ganswindt, Adrian S. W. Tordiffe, Heribert Hofer, Martin Dehnhard

**Affiliations:** 1 Department Reproduction Biology and Evolutionary Ecology, Leibniz Institute of Zoo and Wildlife Research, Forschungsverbund Berlin e.V., Berlin, Germany; 2 Endocrine Research Laboratory, Department of Anatomy and Physiology, Faculty of Veterinary Science, University of Pretoria, Onderstepoort, Republic of South Africa; 3 National Zoological Gardens of South Africa, Pretoria, Republic of South Africa; 4 Department of Companion Animal Clinical Studies, Faculty of Veterinarian Science, University of Pretoria, Onderstepoort, Republic of South Africa; Baylor College of Medicine, UNITED STATES

## Abstract

Enzyme immunoassays (EIA) that measure faecal testosterone metabolites (fTM) are useful tools to monitor gonadal activity. The aim of this study was to validate an “in-house” epiandrosterone EIA to monitor fTM in spotted hyenas. FTM were characterised in a male and a female hyena that each received an injection of ^3^H-testosterone. High-performance liquid chromatography (HPLC) analyses revealed a cluster of highly polar enzyme-hydrolysable hormone metabolite conjugates. We performed hydrolysis using β-glucuronidase to deconjugate metabolites and improve sensitivity of the assay. Because β-glucuronidase from *Helix pomatia* has been reported to bias testosterone measurements in some species, we compared the enzymatic activity of the commonly used β-glucuronidase extracted from *H*. *pomatia* with the same enzyme from *Escherichia coli*. Our results showed that β-glucuronidases from both sources produced similar results from spotted hyena faeces. We therefore hydrolysed samples with *H*. *pomatia* enzymes. HPLC analyses also demonstrated that following hydrolysis the epiandrosterone EIA measured significant amounts of immunoreactive metabolites corresponding to radiolabelled metabolites in both sexes. Additionally, HPLC and GC-MS analyses confirmed the presence of epiandrosterone in faeces of spotted hyenas. The biological relevance of the epiandrosterone EIA was validated by demonstrating (1) a significant increase in fTM levels in response to a testosterone injection within 16 h, (2) no biological responsiveness to an adrenocorticotropic hormone (ACTH) injection and (3) significant differences in fTM levels between juvenile males and adult immigrant males in a free-ranging wild population. Our results clearly demonstrate that the epiandrosterone EIA is a reliable non-invasive method to monitor gonadal activity in spotted hyenas.

## Introduction

In mammals, androgens are involved in shaping phenotypic and life history traits[1, 2]. Androgens influence gene expression and the development of foetuses, secondary sexual characteristics and sexually dimorphic behaviour [3, 4]. Contest competition among males for access to mating partners can be associated with elevate androgen concentrations (e.g. [5, 6–8]), as predicted by the ‘challenge hypothesis’ [9]. Similarly, in some species contests among males for social dominance can results in dominant males having higher circulating concentrations of androgens than subordinate males (e.g. African wild dog, *Lycaon pictus*, [10]; golden lion tamarin, *Leontopithecus rosalia*, [7]), but this is not the case in other species (e.g. common brown lemur, *Eulemur fulvus rufus*, [11]; bonobo, *Pan paniscus*, [8]).

Routinely, blood samples are used to measure circulating hormone concentrations to evaluate an animal’s endocrine status. However, restricted access to animals may limit blood sampling, particularly in wildlife species [12]. Furthermore, handling procedures including capture, restraint and anaesthesia, which are necessary for blood sampling, may alter circulating hormone concentrations, which is especially problematic when an individual is handled repeatedly to obtain multiple samples [13–15]. The measurement of faecal hormone metabolite concentrations using enzyme immunoassays (EIA) provides a non-invasive alternative method to the use of blood samples. Faecal samples can be collected easily and without disturbing the animal, thereby allowing repeated sampling, even over long periods [12, 15]. Furthermore, faecal hormone metabolite concentrations are integrated over a period of time and hence are less affected by the pulsatory hormone secretion pattern present in blood samples. Hence the application of EIAs has many advantages—but their application presents several challenges.

Circulating steroid metabolites are extensively metabolised by the liver and intestinal bacteria, thus faecal steroid metabolites are a mixture of several metabolites [16]. Therefore, EIAs used to monitor faecal hormone metabolites and extraction procedures for steroids from faeces should be carefully validated for each species and type of hormone [16–18]. Antibodies used for EIAs are group-specific and rely on cross-reactivities to hormone related metabolites with similar functional groups present in the native steroid hormone [15]. Steroid hormones, such as androgens, may be converted to polar water-soluble glucuronide and/or sulphate conjugates prior to excretion [15], which have been demonstrated by radiometabolism studies to occur in the faeces of a taxonomically diverse range of mammalian species (e.g. primates [19]; maned wolf, *Chrysocyon brachyurus* [20]; Eurasian lynx, *Lynx lynx*, and Iberian lynx, *Lynx pardinus* [21]; spotted hyena, *Crocuta crocuta* [22]). Hydrolysis can liberate steroids from their conjugates, thereby increasing steroid detection by antibodies and thus the sensitivity of EIAs. The enzyme β-glucuronidase from *Helix pomatia* is commonly used to deconjugate steroids, but its application may transform androgens and produce the artefact of an increase in the “measured” testosterone levels [23–26]. One known major challenge to the development of an EIA for measuring faecal androgen metabolites (fAM) is that the majority of faecal androgens and some of the faecal glucocorticoid metabolites (fGM) have similar androstane-based structures, hence antibodies directed against C_19_-cortisol metabolites may cross-react with androgen metabolites [15, 27].

Our study aimed to validate an “in house” EIA to monitor faecal testosterone metabolites (fTM) in the spotted hyena, suitable for monitoring gonadal activity in wild populations. The spotted hyena is a highly social carnivore (for a review of the species biology see [28]). Spotted hyenas live in groups termed clans [29] containing natal females, their juvenile offspring and reproductively active adult males that are mostly but not exclusively immigrants from other clans [30, 31]. One noticeable feature of spotted hyena society is the low frequency and low intensity of physical aggression among immigrant males [30, 32]. At immigration, males join the male dominance hierarchy at the lowest rank and increase in dominance as males of higher status die or leave the clan [30]. Hence the immigrant male hierarchy functions as a social queue because males mostly adhere to a strict queuing convention [30]. The rank of an immigrant male is therefore chiefly determined by his length of tenure [30].

All female clan members breed and births occur throughout the year [33, 34], yet high-ranking males do not monopolise the paternity of offspring produced in a clan [35]. Rather, females exercise strong mate-choice [31, 35] because the occurrence and position of their peniform clitoris [36, 37] ensures that copulation cannot occur without the complete cooperation of the female [29, 32]. Immigrant males employ various mating tactics [30, 35, 38]. One tactic used mostly by immigrant males above median rank is to prevent lower-ranking immigrant males interacting with the female they have chosen to “defend” [30]. In accordance with the ‘challenge hypothesis’ [9] immigrant males defending females have higher testosterone concentrations than those not defending females [5]. Furthermore, because of the typically low frequency and intensity of aggression among immigrant males there is no significant correlation between an immigrant male’s rank and his testosterone concentration [5, 39]. In general, testosterone concentrations in young pre-dispersal natal males are lower than those of immigrant males, and normally remain low for approximately 12 months after dispersal and immigration into another clan [5, 40].

In this study we: (1) investigated the efficiency of an EIA with an antibody directed against epiandrosterone (epi-A) to monitor the expected increase in fTM following the injection of 20 mg testosterone in a female captive hyena; (2) characterised faecal testosterone metabolites by performing a radiometabolism study and evaluating the specificity of the epi-A antibody by comparing immunoreactivities towards radiolabelled peaks originating from testosterone degradation using HPLC analyses; (3) identified the major fTM by gas chromatography–mass spectrometry (GC–MS) analyses; (4) assessed whether enzymatic hydrolysis with β-glucuronidase extracted from either *H*. *pomatia* or *E*. *coli* influenced our measurements of fTM concentrations; (5) determined whether our EIA was influenced by fGM of adrenocortical origin by comparing levels of fGM and fTM in samples of an ACTH challenge test and (6) biologically validated the epi-A EIA by measuring fTM concentrations of free-ranging juvenile natal male and adult immigrant male spotted hyenas, with the expectation that juveniles would have lower fTM levels than adult immigrant males.

## Material and Methods

### Ethics Statement

The treatment of the captive spotted hyenas was performed when the animals were anaesthetised by zoo veterinarians using a combination of medetomidine, midazolam and butorphanol at the National Zoological Gardens (NZG) of South Africa and ketamine hydrochloride and xylazine at Leipzig Zoo. All methods applied, and the study design were approved and in agreement with the animal ethics and welfare committee at the Leibniz Institute for Zoo and Wildlife Research (IZW), the Leipzig Zoo and NZG (permit numbers: 2012-03-01, Leipzig Zoo, Germany; 2012-08-31, NZG of South Africa). The field research and collection of faecal samples of free-ranging spotted hyenas in Tanzania was approved by the Tanzanian Commission for Science and Technology (COSTECH) and the Tanzania Wildlife Research Institute (TAWIRI). The permission to work in the Serengeti National Park was granted by Tanzanian National Parks Authority (TANAPA).

### Study animals and processing of faecal samples

#### Sample collection in captivity

One adult female and two adult male spotted hyenas were studied in captivity. The female was kept in the National Zoological Gardens of South Africa, Pretoria, whereas the males were kept in Leipzig Zoo (Germany) and Dvur Králové Zoo (Czech Republic), respectively. All faecal samples were collected by animal keepers within the animal management schedule of each zoo. The samples were taken from the centre of a dropping to avoid cross-contamination with urine. The samples were mixed by hand and a 5–10 g portion was placed in individual collection vials and frozen immediately upon collection at -20°C.

#### Sample collection in the wild

A total of 32 faecal samples were collected immediately after deposition from individually known free-ranging spotted hyenas in the Serengeti National Park in northern Tanzania (adult immigrant male hyenas, n = 15; adult females, n = 1; juvenile males n = 15). To avoid the potential impact of twin litter sibling aggression [41–43] on fTM levels we only analysed faecal samples from singleton male litters. Faeces were stored in the field in a cold box for less than 3 hours after collection. Faeces were mechanically mixed and an aliquot of faeces was stored in either liquid nitrogen or frozen at approximately -10°C in Tanzania until transported frozen to Germany where they were stored at -80°C until analyses.

The animals were members of three clans which are part of a long-term research programme [35]. Animals were categorised as juveniles when less than 24 months of age and as adults thereafter [44, 45]. All samples from adult males were from immigrant males with at least 12 months of tenure. We recognised individuals by their distinctive spot patterns, ear notches, scars and bald patches [42, 46]. The age of juveniles was estimated in days when first observed at the birth den or the communal den using pelage characteristics, whether their ears were flattened or upright, and their balance and coordination during locomotion [47]. Estimates of age were accurate to within 7 days. We used the dimorphic glans morphology of the erect phallus to determine sex [48].

#### Extraction of faecal samples

For captive spotted hyenas, an aliquot of 0.5 g wet faeces were extracted with 4.5 ml 90% methanol for 30 min using a universal shaker (SM-30, Edmund Buhler GmbH, Hechingen, Germany). After centrifugation (15 min, 1000 g) the supernatant was transferred to a new tube and diluted 1:1 with water. Aliquots of faecal extracts were subjected either to HPLC analyses, or directly to the in-house epiandrosterone EIA.

Faecal samples of free-ranging spotted hyena may vary considerably in their degree of humidity. To avoid a possible diluting effect, faecal samples were dried for 22 h in a freeze-drier (EPSILON1-4, LSC plus, Martin Christ GmbH, Osterode, Germany). After powdering the dried faeces, 0.1 g of well-mixed powder were extracted with 0.9 ml 90% methanol and homogenised on a shaker for 30 min. After centrifugation (3000 rpm, 15 min) the supernatant was transferred to a new tube. For EIA analyses aliquots were diluted 1:2 with water and 20μl were subjected to the androgen EIA.

### Radiometabolism study

To characterise fTM, we performed two radiometabolism studies on one captive adult female and on one captive adult male hyena, respectively.

The radiometabolism study in the female was conducted in combination with a testosterone challenge experiment (see section Testosterone challenge) in the National Zoological Gardens of South Africa (NZG), Pretoria, in 2012. The design of this study followed a standard procedure previously described for hyenas and used for the evaluation of faecal glucocorticoid metabolite measurements [45]. The hyena was immobilized in its enclosure via remote injection (DanInject darting system), using a combination of medetomidine (0.03 mg/kg) + midazolam (0.15 mg/kg) + butorphanol (0.2 mg/kg). After the collection of a blood sample (5 ml in an EDTA tube) taken from the jugular vein, a solution (0.5 ml) containing 20 mg unlabelled and 250μCi ^3^H-testosterone (70–105 Ci/mmol, TRK921, Amersham Bioscience, UK) in ethanol was added to 2.5 ml of a sterile 0.9% NaCl solution, vigorously vortexed, and injected i.m. Faecal samples were collected from 4 days prior to steroid administration until 196 h post-injection.

The radiometabolism study in the male hyena was conducted at Leipzig Zoo (Germany) in 2008. 2.5 ml of a sterile 0.9% NaCl solution was added to a solution (0.25 ml) containing ~250 μCi 1,2,6,7-^3^H-testosterone (70–105 Ci/mmol, TRK402, Amersham Bioscience, UK) in ethanol and injected into the cephalic vein following anaesthesia using a combination of medetomidine (0.03 mg/kg) + midazolam (0.15 mg/kg) + butorphanol (0.2 mg/kg). Faecal samples were collected from 2 days prior to steroid administration until 2 days post-injection.

Aliquots of each sample were extracted for testosterone metabolite determination and radioactivity counting. All radioactive counting was conducted in a Packard TRI-CARB 1900 TR liquid scintillation counter (Canberra-Packard GmbH, Germany). Samples used for HPLC analyses were in the female, the first sample collected 15.5 h following injection, and in the male the sample collected 47.25 h following injection, as they contained the highest amount of radioactivity (see section HPLC analysis), respectively.

Prior to HPLC, both faecal extracts were subjected to enzyme hydrolysis (see below) in order to test whether faecal testosterone metabolites were conjugated to glucuronides.

### Hydrolysis

For hydrolysis, 100μl of faecal extracts were hydrolysed with 900μl 0.05 M acetate buffer (pH 4.8) containing 4μl β-glucuronidase and arylsulfatase from *H*. *pomatia* (*Hp*) (Roche Diagnostics GmbH), hereafter termed *Hp*-enzymes, for 2 h at 37°C. Steroids were then extracted 3 times with 2.5 ml *tert* methylbutylether/petroleum ether (30:70, v/v) for 30 min using a universal shaker. Phase separation was achieved by freezing for 15 min at -80°C. The extracts were combined, evaporated in a sample concentrator (Dri Block DB3, Techne, Staffordshire, UK) under nitrogen at 55°C, dissolved in 40% methanol and stored at -20°C until HPLC analyses.

Hydrolysis with β-glucuronidase from *E*. *coli* (*Ec*), hereafter termed *Ec*-enzyme, was modified from [23]. Lyophilized β-glucuronidase Type VII-A from *E*. *coli* (5000U, Sigma-Aldrich, Inc. USA) was dissolved in 1.4 ml water. 200μl of faecal extracts were diluted in 800μl 0.25 M phosphate buffer (pH 6.9) and then hydrolysed adding 40μl enzyme for 22 h at 37°C. Steroids were extracted twice with 3 ml *tert* methylbutylether/petroleum ether (30:70, v/v), for 30 min using a universal shaker. Phase separation was achieved by freezing for 15 min at -80°C. The extracts were combined, and processed as described above.

To compare the enzymatic activity of β-glucuronidase from *Hp* and *Ec*, faecal extracts from the testosterone challenge were hydrolysed with both enzymes. Because both enzymes produced highly congruent results, hydrolysis of all further samples was performed using β-glucuronidase from *Hp*.

### HPLC analysis of faecal testosterone metabolites

Aliquots of 150μl faecal extracts of non-hydrolysed and hydrolysed samples from one female and one male hyena containing the highest amount of radioactivity, respectively and two hydrolysed samples containing high endogenous concentrations of epiandrosterone from one free-ranging male and one free-ranging female hyena, respectively, were selected for HPLC analyses. Sample preparation on C_18_ columns and chromatographic separation using an Ultra Sep ES-RP 18/6 μm HPLC column (250x4 mm, Sepserv, Berlin, Germany) were carried out as previously described [49]. The elution positions of authentic cortisol, corticosterone, testosterone, epiandrosterone and dihydrotestosterone (DHT) on this column were determined in separate HPLC runs.

### Testosterone challenge

The testosterone challenge experiment was conducted in combination with a radiometabolism study (see section Radiometabolism study) in the female hyena in 2012. The hyena received 20 mg unlabelled testosterone. In addition to faecal samples, one blood sample prior to and after the injection was taken to prove the increase in plasma testosterone. Following extraction, faecal samples were subjected to enzymatic hydrolysis and stored at -20°C until analyses.

### ACTH challenge

The ACTH challenge was conducted in Dvur Králové Zoo (Czech Republic) in 1998 on a male captive adult spotted hyena. Detailed descriptions of treatment and sample collection procedures were previously published [45, 50].

### Enzyme immunoassays

Methanol-extracted faecal samples were analysed with an “in-house” epiandrosterone (epi-A) EIA (endocrinology laboratory of the IZW). The antibody was polyclonal and was raised in rabbit against the 3-hemisuccinat (HS)-steroid coupled with bovine serum albumin (BSA). The corresponding epiandrosterone-3-HS-peroxidase was used as label. The antibody and label were used in a 1:20.000 and 1:2.000 dilution, respectively. The cross-reactivities of the antibody were determined as follows: 100% epiandrosterone (5α-androstan-3β-ol-17-one), 88% epiandrosterone glucuronide (5α-androstan-3β-ol-17-one glucosiduronate), 60% androsterone (5α-androstan-3α-ol-17-one), 60% androstenedione (4-androsten-3, 17-dione), 24% androsterone sulphate (5α-androstan-3α-ol-17-one sulphate), 19.2% dehydroandrosterone (5-androsten-3α-ol-17-one), 17% dehydroepiandrosterone (5-androsten-3β-ol-17-one), and 0% for cortisol and corticosterone. The assay was validated by demonstrating parallelism of faecal extracts to the epiandrosterone standard curve. The intra-assay and inter-assay coefficients of variation were determined by using faecal extracts containing known concentrations of epi-A. The inter-assay coefficients were 8.6% (n = 10) for extracts containing low and 9.3% (n = 10) for extracts containing high concentrations of epi-A. The intra-assay coefficients were 12.7% (n = 8) for extracts containing low and 6.7% (n = 8) for faecal extracts containing high concentrations of epi-A.

Testosterone measurements in blood plasma (see section Testosterone challenge) were carried out using an antibody directed against testosterone-11-HS-BSA as previously described for blood plasma [51]. For comparative measurements, faecal samples from the ACTH challenge were also analysed using an antibody directed against cortisol-3-CMO-BSA, previously validated to assess changes in adrenocortical activity in spotted hyenas based on fGM analyses [45], and an antibody directed against testosterone-11-HS-BSA, previously validated for fTM analyses in lynx [21]. Enzyme immunoassays were performed as previously described [49].

### Gas chromatography-mass spectrometry (GC–MS)

GC-MS analyses were carried out as previously described [49] following derivatisation of samples and standards with N-methyl-N-trimethylsilyl-trifluoroacetamide (MSTFA, CS-Chromatographie Service, Langerwehe, Germany), trimethylsilyl iodide (TMSI), and dithioerythritol (DTE, both from Sigma Chemie GmbH, Deisenhofen, Germany) according to Magnisali et al. (2008) allowing quantification of the resulting trimethylsilyl derivatives by GC–MS. Sample analysis was performed on an Agilent 7890A Gas Chromatograph (Agilent Technologies, Böblingen, Germany) equipped with an Agilent HP-5MS (5% phenyl-, 95% methylsiloxane) fused silica capillary column (30 m x 0.25 mm i.d. x 0.25μm) interfaced with an Agilent 5975C mass selective detector.

The MS was operated in the EI mode with the electron voltage set to autotune value. MS acquisition was performed in selected ion monitoring mode (SIM) by monitoring the ions m/z 434 and 419 for epiandrosterone. The Agilent MSD chemstation data chemstation software was used for peak integration and library searches.

### Data analysis

Calculation of baseline values was performed using an iterative process excluding all values greater than the mean +2SD [52]. Significant increases were defined as peaks with values exceeding the baseline +2SD. Statistical analyses were performed with the NSM3 package version 1.1 in R version 3.0.2 [53]and StatXact version 10 (Cytel Software Inc., Cambridge, Massachusetts, USA). The level of significance was fixed at 5% and all tests were two-tailed. For biological validation of the epiandrosterone EIA we compared fTM levels between juvenile male and adult immigrant male spotted hyenas. Since the concentrations in both groups differed in dispersion, as documented by Conover’s squared ranks test [54] (T = 2559, exact p = 0.0019), the usual procedure of applying a Mann-Whitney U-test would have been inappropriate. Instead, we resorted to two robust tests which do not make assumptions about equality of dispersion, the Fligner-Policelli test [55] and the permutation test [56] in the version of the maximin efficiency robust test approach of Gastwirth [57], as implemented in StatXact.

## Results

### Radiometabolism study and HPLC analyses

**Fig 1:**
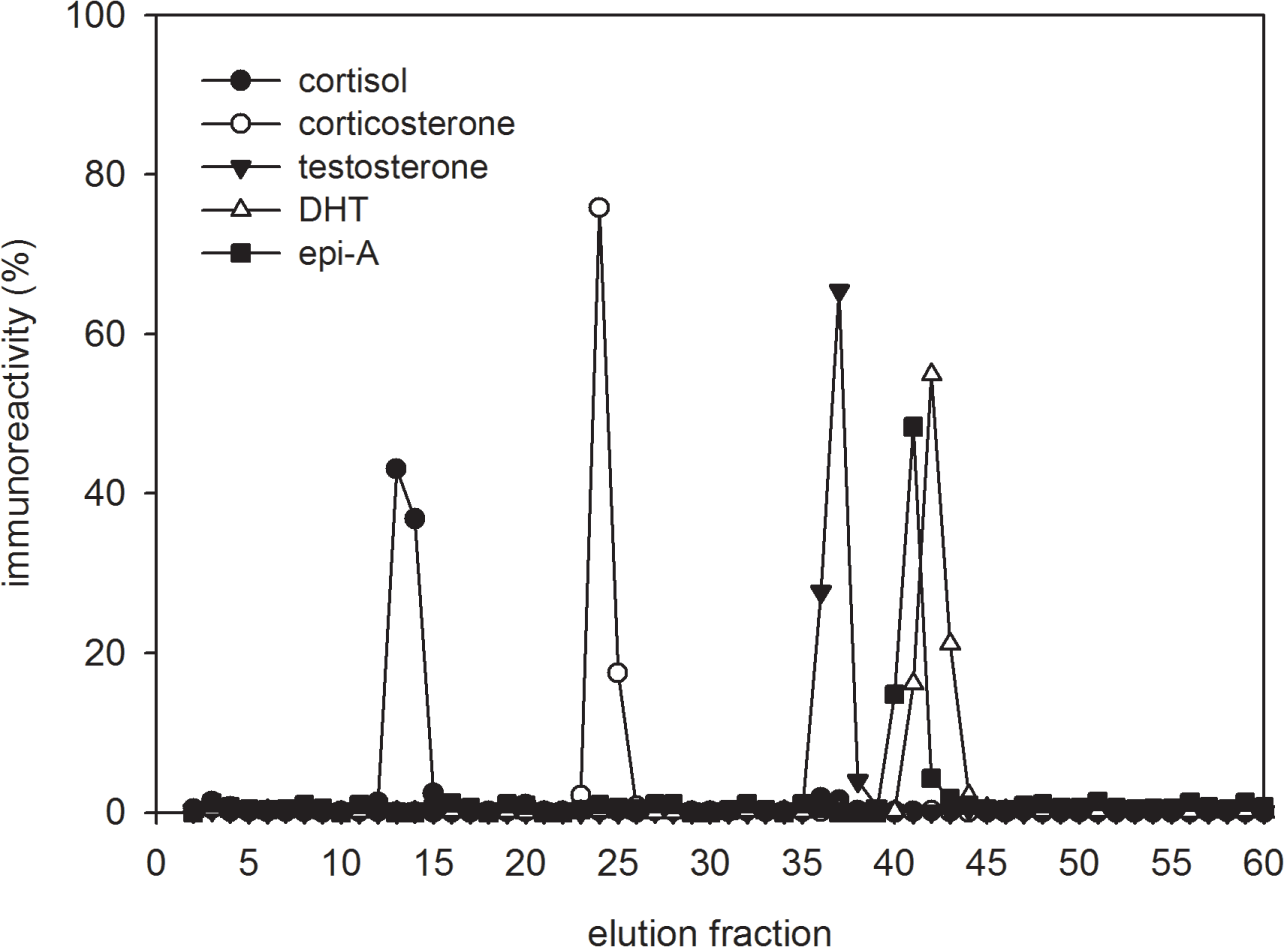
HPLC profiles of steroid standards. Elution positions of authentic cortisol, corticosterone, testosterone, epiandrosterone and dihydrotestosterone (fractions 12, 23, 36, 40, and 41 respectively) obtained by applying the corresponding steroid hormone specific assays. For comparison results are presented as percentage of overall eluted steroid concentration.

**Fig 2:**
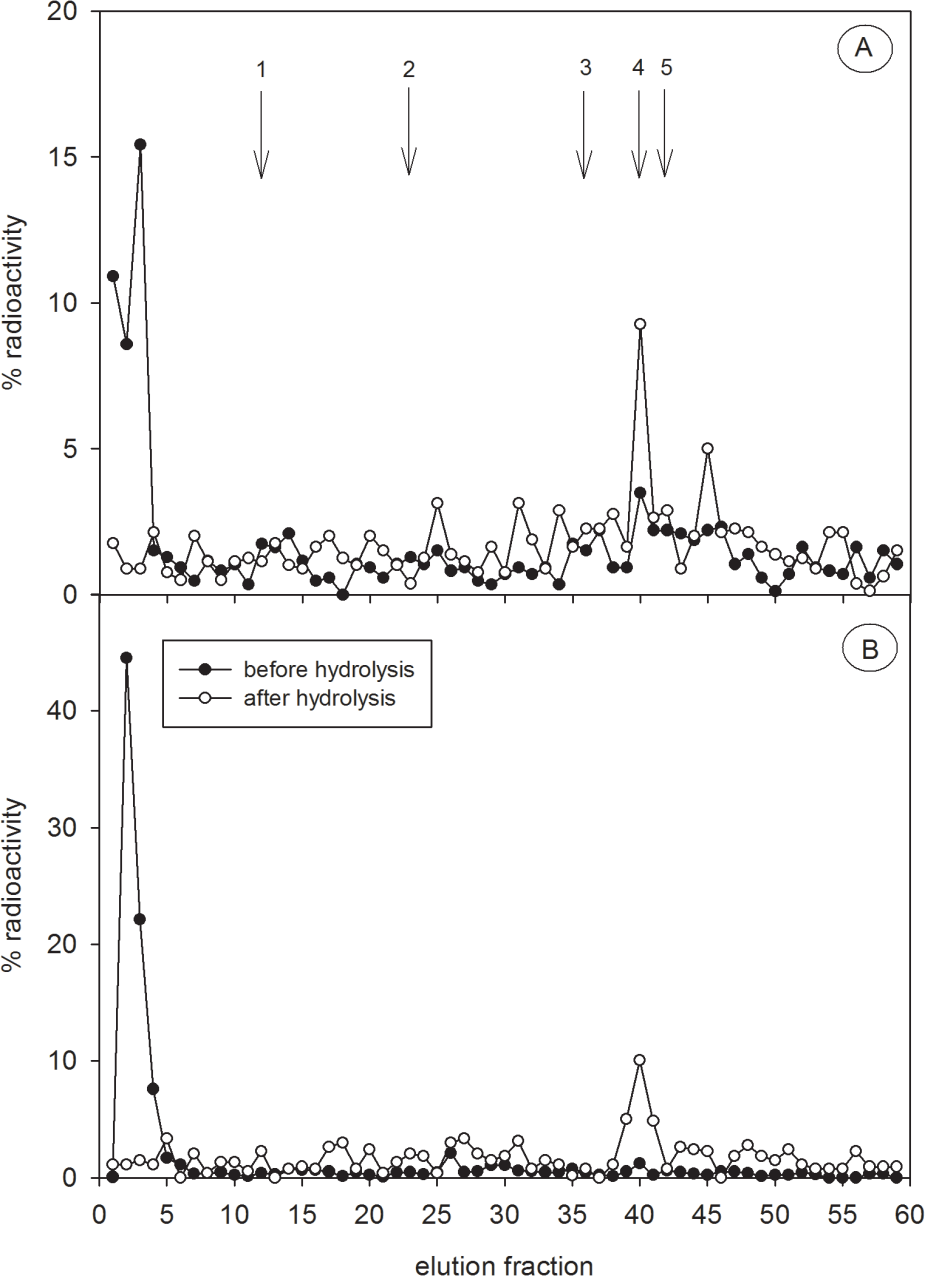
HPLC profiles of ^3^H-testosterone metabolites. 3H-testosterone metabolites were analysed in non-hydrolysed (black circles) and hydrolysed (white circles) faecal extracts of one captive male (A) and one captive female (B) spotted hyena. Extracts were separated by RP-HPLC and then radioactivity of each fraction was analysed. Radioactivity is presented as a percentage of the overall eluted activity. The arrows represent the elution positions of reference standards cortisol (1), corticosterone (2), testosterone (3), epiandrosterone (4) and dihydrotestosterone (5), as detailed in Fig 1.

HPLC elution positions of the reference steroid standards cortisol, corticosterone, testosterone, dihydrotestosterone and epiandrosterone obtained when applying the corresponding steroid hormone specific assays are shown in Fig 1. HPLC profiles of radiolabelled testosterone metabolites in non-hydrolysed and hydrolysed spotted hyena faeces are shown in Fig 2A&2B, respectively. Using reversed-phase HPLC, metabolites were separated according to their polarity and the more polar metabolites were eluted first. In non-hydrolysed extracts of one captive adult male (Fig 2A) and captive adult female (Fig 2B) hyena, only one polar peak was detected in fractions 1–5, probably indicating a cluster of conjugated radiolabelled metabolites. Minor variations in the composition of radiolabelled metabolites in both sexes might result of individual or sex-specific differences in testosterone metabolism. Enzymatic hydrolysis of faecal extracts changed the elution patterns of polar radiolabelled metabolites in extracts from both sexes. In the male (Fig 2A) as well as in the female (Fig 2B) polar radiolabelled conjugates disappeared and were substituted by a major radiolabelled peak in fractions 39–41, co-eluting with the epi-A standard. In the male an additional minor proportion of radiolabelled metabolites occurred in fractions 44–46. In both sexes no radiolabelled metabolites were detectable at the elution position corresponding to the testosterone standard, thus the circulating hormone itself is not present in faeces. Additionally, no radiolabelled metabolites were detected at the elution positions of authentic cortisol, corticosterone and DHT.

**Fig 3:**
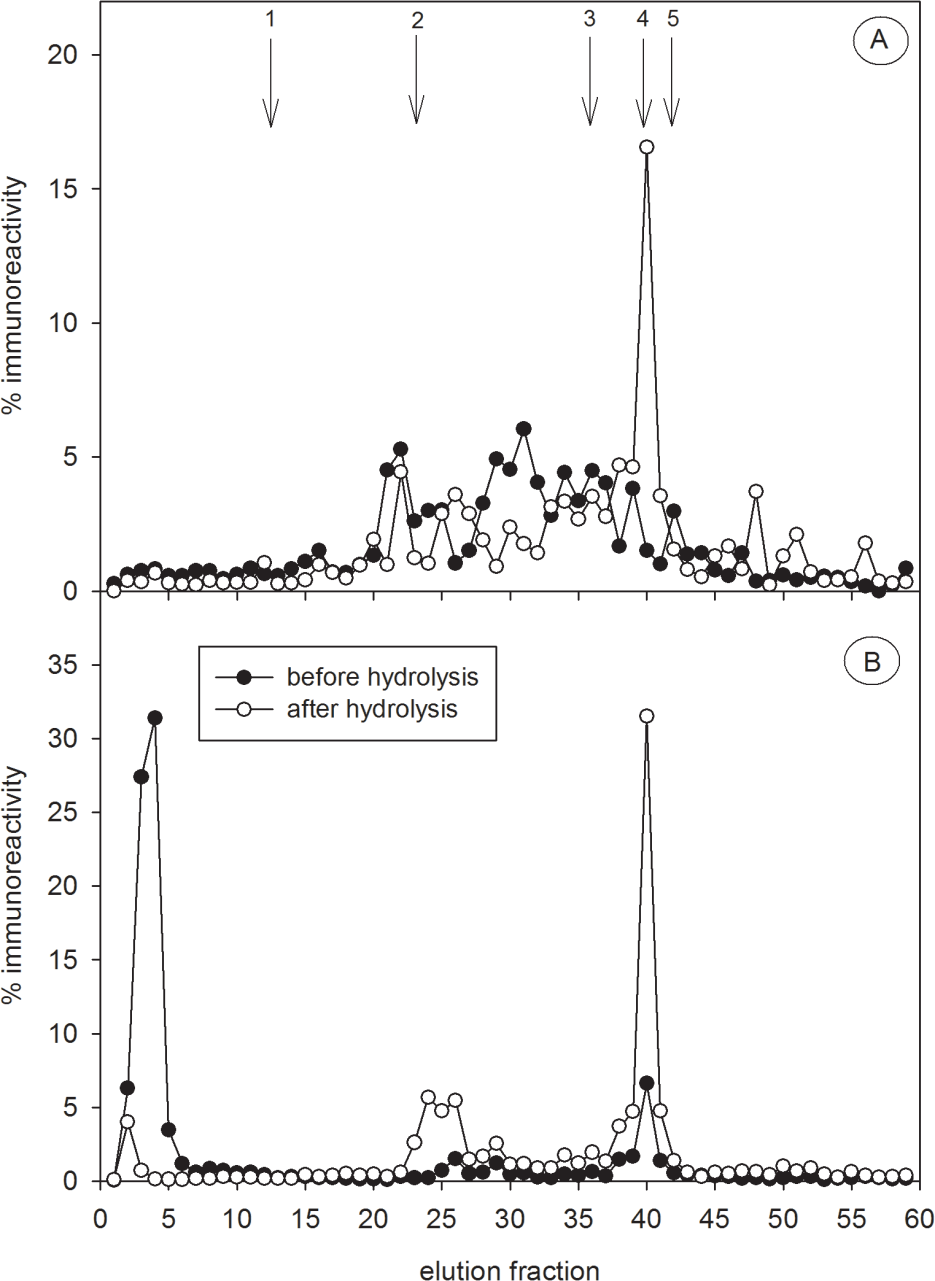
HPLC profiles of immunoreactive testosterone metabolites in captive hyenas. Testosterone immunoreactivity of faecal extracts were analysed in faecal extracts of one captive adult male (A) and one captive adult female (B) spotted hyena. Immunoreactivity was determined in the epiandrosterone EIA and is presented as a percentage of overall eluted activity. Lines with black circles represent immunoreactivity in each fraction. Lines with white circles show immunoreactivity in the fractions of the same extract after hydrolysis. The arrows represent the elution positions of reference standards cortisol (1), corticosterone (2), testosterone (3), epiandrosterone (4) and dihydrotestosterone (5).

**Fig 4:**
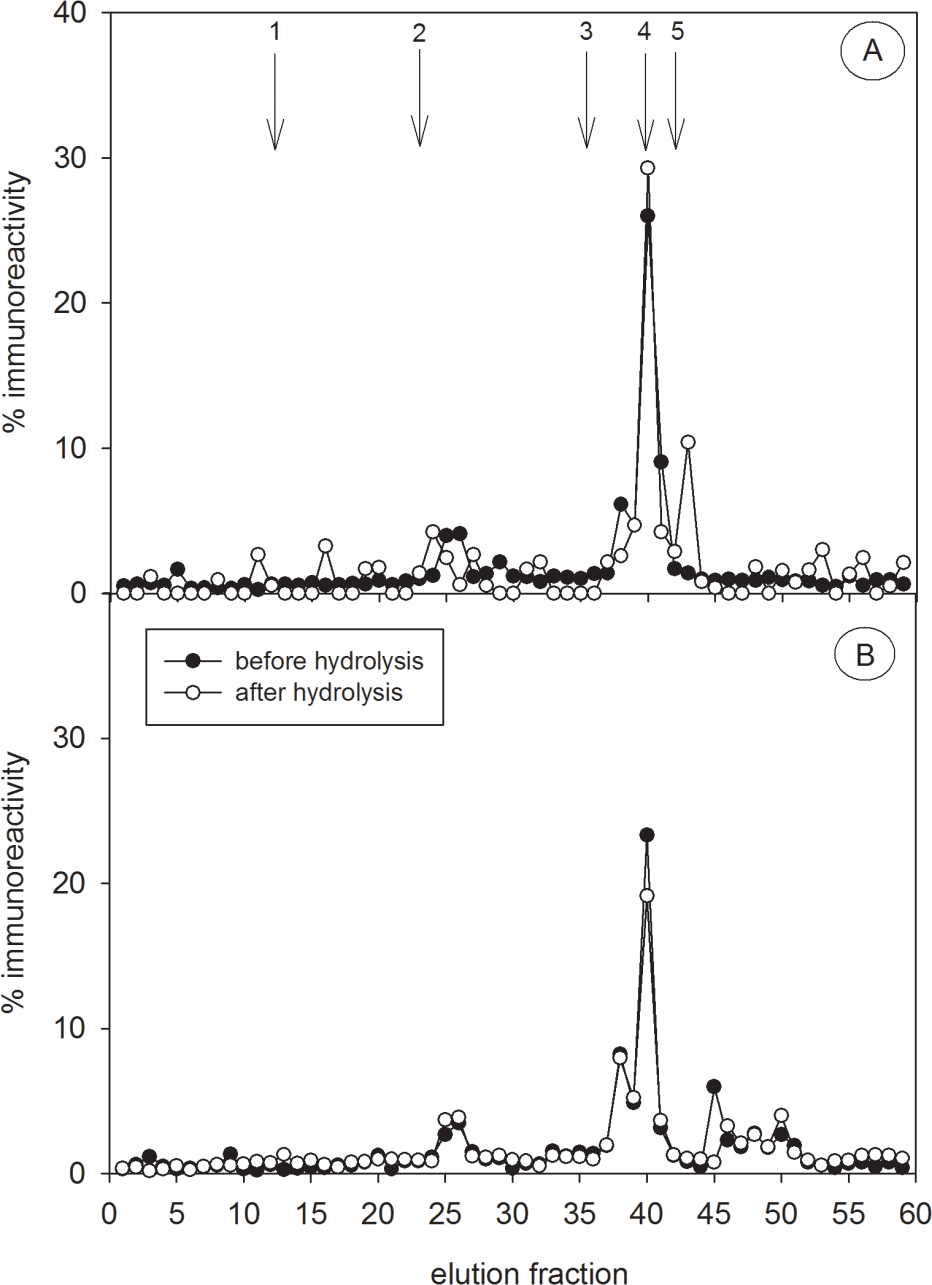
HPLC profiles of immunoreactive testosterone metabolites in free-ranging hyenas. Testosterone immunoreactivity was analysed in faecal extracts of one free-ranging adult male (A) and one free-ranging adult female (B) hyena. Immunoreactivity was determined in the epiandrosterone EIA and is presented in percentage of overall eluted activity. Lines with black circles represent immunoreactivity in each fraction. Lines with white circles show immunoreactivity in the fractions of the same extract after hydrolysis. The arrows represent the elution positions of reference standards cortisol (1), corticosterone (2), testosterone (3), epiandrosterone (4) and dihydrotestosterone (5).

Before hydrolysis, HPLC profiles revealed polar radiolabelled testosterone metabolites (Fig 2) that were not apparent in the HPLC immunograms of the captive male (Fig 3A), the free-ranging adult male (Fig 4A) and the free-ranging adult female (Fig 4B), probably due to the inability of the polar radiolabelled metabolites to cross-react with the antibody. In contrast, before hydrolysis the polar radiolabelled metabolites from the captive female (Fig 2B) fitted with corresponding polar immunoreactivities (Fig 3B). Nevertheless, hydrolysis with the *Hp*-enzymes standardizes the patterns of immunoreactivities in both the captive male and female (Fig 3), liberating a major metabolite from its conjugate corresponding to the elution position of epi-A in fraction 40 and a minor one at fraction 25 (Fig 3). Similarly, in the hydrolysed extracts from an adult free-ranging male (Fig 4A) and an adult free-ranging female (Fig 4B), the epi-A EIA demonstrated a major peak of immunoreactive metabolites in fractions 40, co-eluting with the epi-A standard, and two minor peak in fractions 25 and 43, respectively. As a consequence of these results, all further analyses were carried out in hydrolysed extracts. To exclude crossreactivities of the epiandrosterone antibody with other possible faecal steroid metabolites, we analysed the HPLC elution fractions from the captive female with a cortisol-21, corticosterone-21, testosterone and dihydrotestosterone (DHT) EIA, respectively. In comparison to the epi-A EIA, none of the EIAs detected significant amounts of immunoreactivities (S1 Fig). This suggests that additional steroid metabolite levels did not falsify the measurement of immunoreactive epiandrosterone metabolites.

### GC-MS analyses

Prior to analyses of faecal extracts, the retention times of the silylated standard epiandrosterone were established. Following derivatisation of faecal extracts we expected to detect epiandrosterone following hydrolysis with *Hp*-enzymes in samples from the testosterone challenge (see below) by characteristic mass-to-charge ratios (m/z 434 and m/z 419). Applying the SIM modus, we confirmed the presence of epiandrosterone based on its retention time and its m/z ratios of 434 and 419 (434 minus two silylations, 2 x 72, reveals a molecular weight of 290, that of epi-A, data not shown).

### Testosterone challenge

**Fig 5:**
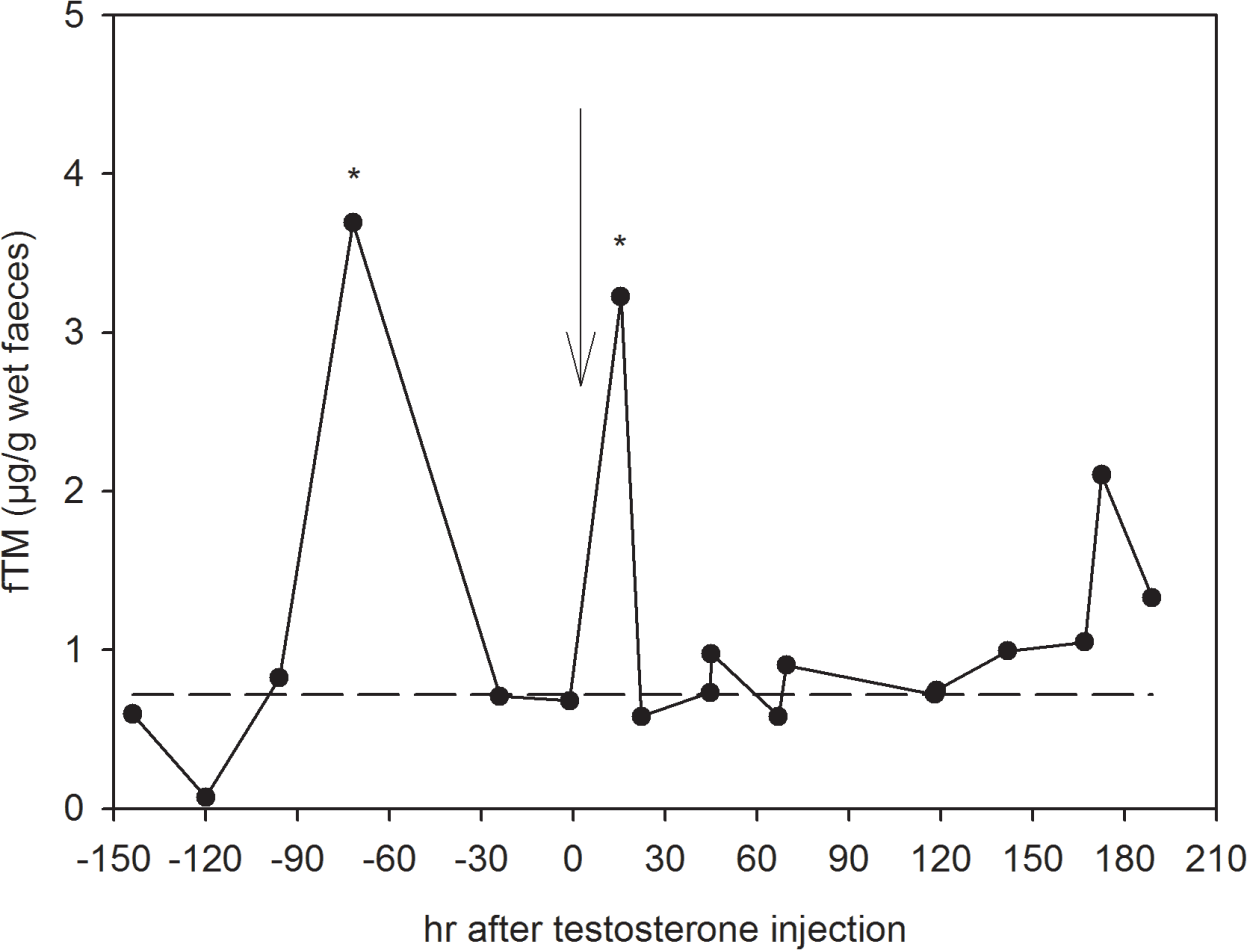
Changes in fTM concentrations in response to a testosterone challenge in a female spotted hyena. Faecal samples were collected from 6 days prior to injection until 8 days post-injection and were analysed with an epiandrosterone EIA following hydrolysis with β-glucuronidase from *Helix pomatia*. The arrow represents the time of testosterone injection; the dashed line indicates the baseline level. The * indicates peaks (values exceeding mean + 2SD).

**Fig 6:**
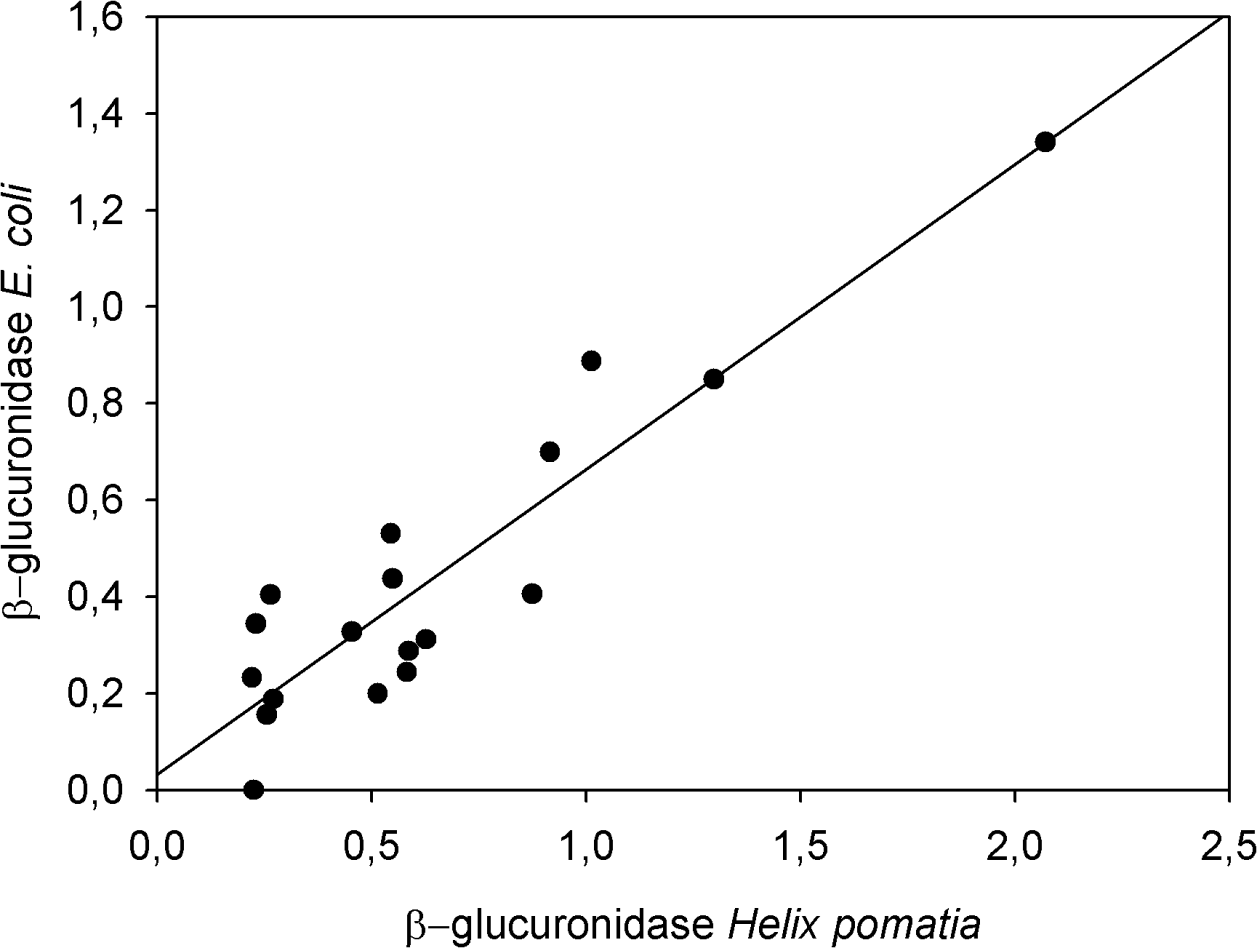
Comparison of testosterone immunoreactivity following hydrolysis with β-glucuronidase from *Helix pomatia* and from *Escherichia coli*. FTM concentrations were determined in faecal samples from the testosterone challenge in the epiandrosterone EIA following hydrolysis with β-glucuronidase from *Helix pomatia* and β-glucuronidase from *Escherichia coli*, respectively. The linear regression indicates that both hydrolysis methods are congruent, as the regression explains a large segment of the variance (r^**2**^ = 0.84) and the intercept does not significantly differ from zero (see text for details).

Administration of testosterone results in a rapid artificial induced increase in plasma testosterone levels and should be clearly reflected in the concentrations of fTM after a species-specific time lag. In the female spotted hyena an increase in concentrations of plasma testosterone from 11 ng/g to 238 ng/g was detected 15 min after injection. To validate the ability of the epi-A EIA to detect the expected increase in fTM caused by the testosterone challenge, we analysed the faecal extracts following hydrolysis with *Hp*-enzymes. A significant increase in fTM levels (Fig 5) was detected 15.5 h post-injection, with a maximum concentration 4-fold above baseline (0.72 ± 0.24μg/g wet faeces). FTM returned to baseline levels within 24 h following injection. This course is congruent with the course of total amounts of radiolabel excreted with faeces. However, a peak of almost a similar intensity was detected 3 days before testosterone treatment. *Ec*-hydrolysed extracts revealed a similar profile of fTM concentrations in response to the testosterone challenge (Fig 6). A linear regression of *Ec*-hydrolysis fTM levels on *Hp*-hydrolysis fTM levels produced a high degree of congruence (r^2^ = 0.83; F = 81.36; df = 1, 16; p < 0.0001, with the regression equation being fTM concentration (*Hp*) = 0.06 + 1.32 x fTM concentration (*Ec*), and the intercept not being significantly different from 0, t = 0.792, p = 0.44). Therefore, all further analyses were carried out in *Hp*-hydrolysed faecal extracts.

### ACTH challenge test

**Fig 7:**
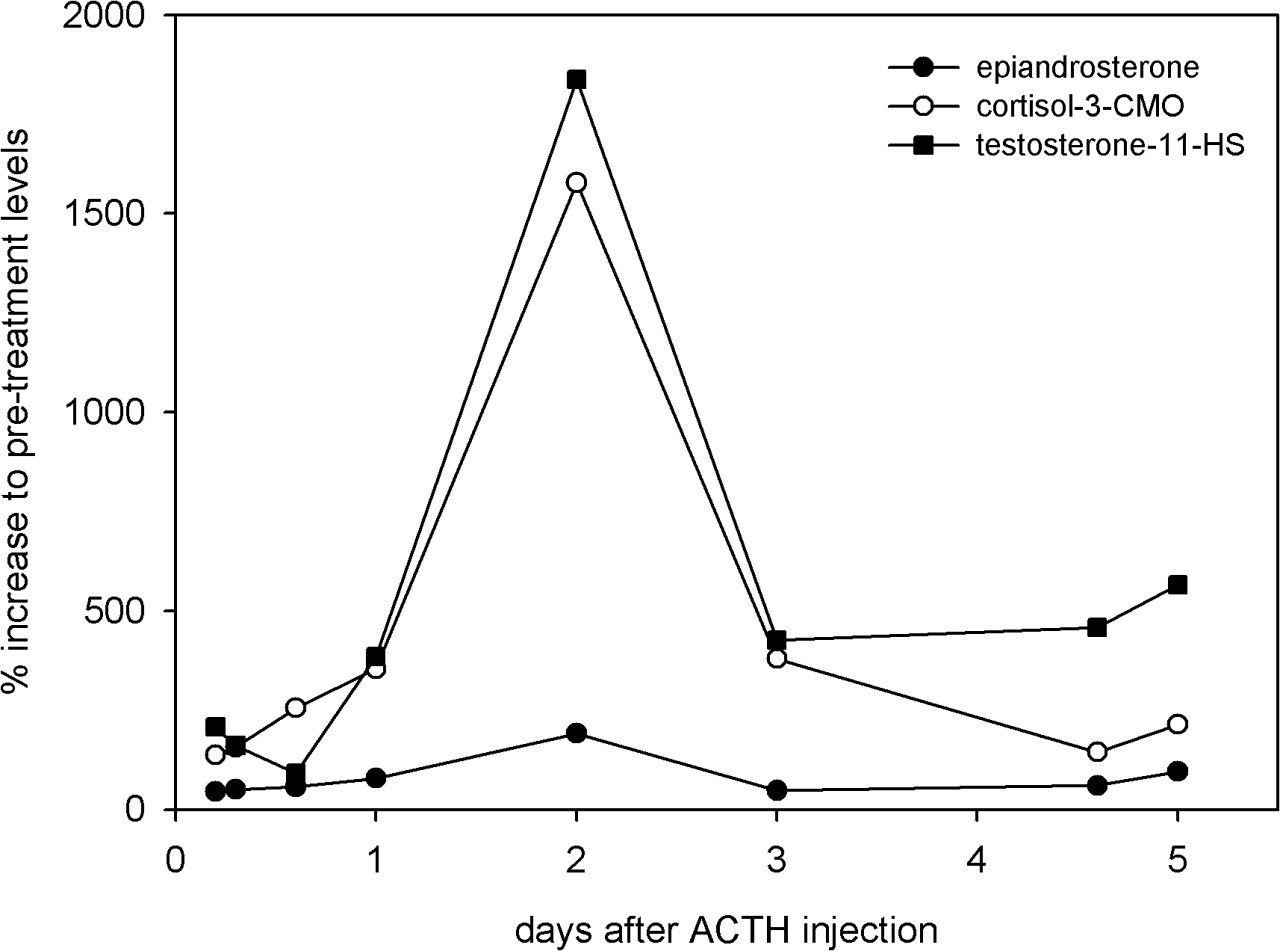
Comparison of faecal cortisol and testosterone immunoreactivity. Changes in fGM and fTM concentrations were determined in faecal samples from the ACTH challenge following hydrolysis with β-glucuronidase from *Helix pomatia* in the cortisol-3CMO, epiandrosterone and testosterone-11-HS EIAs, respectively. Levels of fGM and fTM are shown as percentage increase over pre-injection levels.

To exclude the possibility that the epi-A EIA tracks androgenic steroids from cortisol metabolism, faecal samples from an ACTH challenge experiment were assayed for fGM in hyenas with antibodies directed against cortisol-3-CMO and epi-A EIA (Fig 7), respectively. The cortisol EIA detected a large percentage increase in fGM in response to ACTH, with a maximum concentration 16-fold above pre-treatment levels. No similarly large percentage corresponding increase was obtained when applying the epi-A EIA. This result suggests that androgenic glucocorticoid metabolites do not contribute to the measurement of immunoreactive testosterone metabolites present in faeces of spotted hyenas. In contrast, the use of an EIA with an antibody directed against testosterone-11-HS-BSA revealed that this assay was responsible for substantial cross-reactivities with fGM in faeces from spotted hyenas.

### Comparing levels of fTM in male cubs and adult males

**Fig 8:**
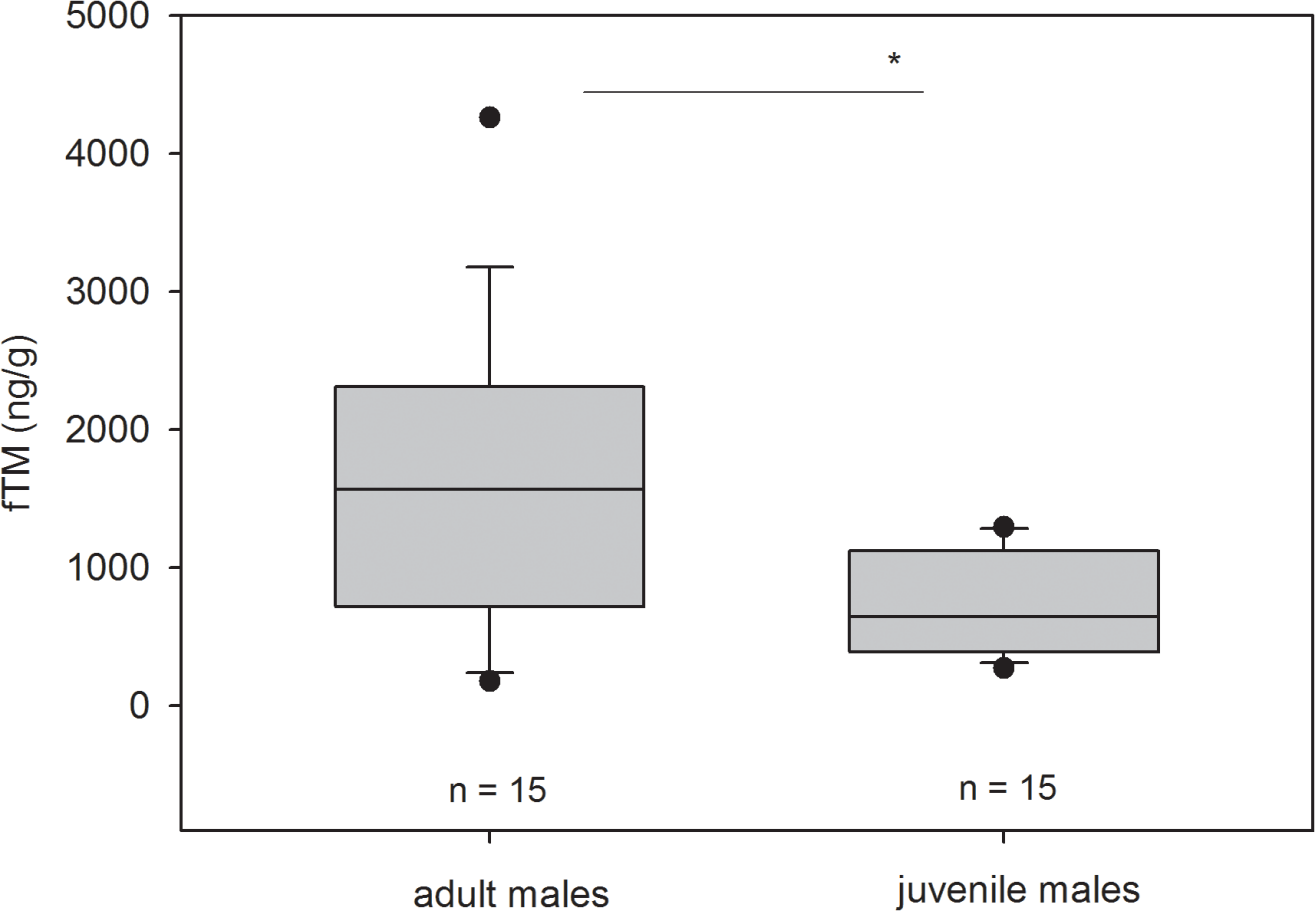
Hormonal data of juvenile and adult immigrant male hyenas. FTM concentrations (ng/g faeces) in juvenile male (n = 15) and adult male (n = 15) spotted hyenas determined in the epiandrosterone EIA following hydrolysis with β-glucuronidase from *Helix pomatia*. The * indicates a significant difference between both categories.

To confirm the physiological importance of faecal testosterone metabolite analyses, we compared fTM levels between adult immigrant male and juvenile male spotted hyenas (Fig 8). Adult immigrant male spotted hyenas possessed significantly higher fTM levels than male juveniles (Fligner-Policelli test, U = 2.3208, exact p using 10,000 Monte Carlo simulations = 0.0294; permutation test, test statistic = -8.349, p = 0.0087). In juvenile males, fTM levels ranged from 272.7 to 1295.05 ng/g wet faeces and in adult immigrant males from 179.3 to 4259.75 ng/g wet faeces, indicating high inter-individual variation.

## Discussion

In this study we demonstrated that an EIA based on an antibody against epi-A is a useful tool for monitoring gonadal activity in spotted hyenas. We detected a significant increase in fTM concentrations following a testosterone challenge experiment. The results of our radiometabolism studies confirmed that the epi-A EIA detected immunoreactive, radiolabelled fTM in both an adult male and female. EIAs using antibodies directed against epi-A have also been validated for monitoring the gonadal status of other mammals, including the African and Asian elephant, *Loxodonta africana* and *Elephas maximus* [58, 59] and the sun bear, *Helarctos malayanus* [60]. Epi-A is also a major testosterone metabolite in faeces of macaques [19, 61]. This suggests our assay may be useful for monitoring fTM across a broader range of mammalian species.

Usually the pattern of radiolabelled metabolites in an HPLC analysis does not coincide with the pattern of immunoreactive hormone metabolites. Part of the reason may be that the antibody used cross-reacts with metabolites other than the targeted radiolabelled metabolites. When interpreting an HPLC immunogram it is extremely important to consider that an immunoreactive peak does not reflect the quantity of faecal hormone metabolites but rather represents the percentage cross-reactivity of the antibody together with the amount of metabolite in a particular HPLC fraction. By contrast, in an HPLC analysis of radiolabelled metabolites, the radioactivity measured in a given elution fraction directly reflects the quantitative amount of the metabolites, even though it cannot be related to any chemical structure [62, 63].

In one captive female, radiolabelled metabolites mainly consisted of polar conjugated metabolites (Fig 2B). Hydrolysis released epi-A from these polar conjugated metabolites regardless of the origin of the glucuronidase enzyme applied (e.g. either from *Hp* or *Ec*). Interestingly in one captive and one free-ranging male and one free-ranging female our epi-A did not detect polar labelled conjugates suggesting possible individual differences in the testosterone metabolism (Figs 3A and 4). Individual differences in steroid metabolisms and those associated with factors such as sex, reproductive status [15] and diet [64] have been documented. Nevertheless, following hydrolysis, profiles of radiolabelled and immunoreactive metabolites were almost identical across the individuals examined (Figs 3 and 4), thereby confirming that testosterone metabolites in spotted hyena faeces are mostly processed via the epi-A pathway and therefore are specifically detected by our epi-A EIA. The antibody might also bind to metabolites not necessarily derived from testosterone degradation, explaining immunoreactivities that are not correlated to radiolabelled metabolites. This might explain the appearance of a minor immunoreactive peak in fraction 25. The presence of epi-A was also clearly confirmed by GC-MS based on its molecular ion of 434 and its retention time on the GC-MS column. In general, our profiles of immunoreactivities differed distinctly from those previously described for fAM (sum of immunoreactive faecal testosterone, DHT and androstenedione metabolites) in spotted hyenas where at least six immunoreactivities covering a wide range of polarities were detected [22]. However, a direct comparison is impossible as solvents of different elution strengths as well as different antibodies were applied.

The results of our radiometabolism studies and comparative HPLC analyses confirmed the presence of epi-A glucuronide as a major conjugate in spotted hyena faeces, with an 88% cross-reactivity with our antibody (see section enzyme immunoassay). The application of hydrolysis increased the accuracy in which our epi-A EIA measured fTM in samples from captive animals (Fig 3), but had little effect on fTM measures in samples from free-ranging animals (Fig 4). Such increased sensitivity is desirable even when only subtle differences in immunoreactivities occur following hydrolysis, because in analyses involving larger samples this will improve the power of statistical comparisons between age or sex categories.

*Hp*-enzymes are commonly used for the total deconjugation of steroids and include both ß-glucuronidase and arylsulfatase activities which can cleave both steroid glucuronides and sulphates. However, several studies have demonstrated that arylsulfatase in *Hp enzymes* cause steroid conversion that increases the measured hormone metabolite concentrations [23, 24]. This unwanted artefact is unlikely to occur with *Ec*-enzymes which do not contain arylsulfatase [24, 25]. Our measurements of immunoreactive fTM following both methods revealed no evidence of this artefact as our *Ec*-hydrolysis produced a similar fTM profile to that following *Hp*-hydrolysis (Fig 6). Furthermore, our HPLC immunograms showed that native testosterone was not present in faeces of spotted hyenas, excluding the possibility that testosterone may have been generated from other metabolites that might have served as precursors for other *Hp*-enzymes [65]. Given that both hydrolysis methods liberated epi-A from its conjugates equally well, our results indicate that in the spotted hyena, the less time consuming *Hp*-hydrolysis can be applied, thereby overcoming the need to implement the complex protocol for *Ec*-hydrolysis [23]. In contrast to *Ec*-hydrolysis, *Hp*-treatment permits a 1:10 dilution with hydrolysis buffer of the faecal epi-A concentrations to reduce the methanol proportion prior to enzyme treatment. Drying down aliquots of faecal extracts and dissolving them in hydrolysis buffer as described earlier in the lynx [21] is not necessary for spotted hyenas.

Structural similarities between androgen and glucocorticoid metabolites [27], can result in cross-reactivities of antibodies directed against androgen metabolites with glucocorticoid metabolites. We examined whether the results of our epi-A EIA included such cross-reactivities using faecal extracts from an ACTH challenge experiment. Glucocorticoid metabolites had no influence on the measurement of fTM (Fig 7), and our HPLC immunograms only reveal significant amount of immunoreactivities which were correlated with radiolabelled fTM. Thus, our results indicate that the epi-A EIA only detected metabolites which derived from testosterone degradation. In contrast, the use of an EIA with an antibody directed against testosterone-11-HS-BSA with spotted hyena faeces, previously validated in two lynx species to detect changes in testicular activity [21], revealed an increase in putative “fTM” almost identical to that measured by the antibody (Fig 7) previously validated to measure fGM in the spotted hyena [45]. Thus, this testosterone antibody is inappropriate to analyse fTM in spotted hyenas because of its substantial cross-reactivities with unknown fGM. This underlines the necessity and importance of validating EIAs for each species and hormone. One minor problem with our EIA were minor immunoreactive peaks between fraction 20 and 28 in captive (Fig 3) and free-ranging (Fig 4) animals which might be caused by non-testosterone metabolites. Even so, this unwanted cross-reactivity is minor compared to the substantial cross reactivities associated with other EIAs (Fig 7).

The first step in our physiological validation of our epi-A EIA revealed a significant increase in fTM levels 15.5 h following injection of unlabelled testosterone in one female spotted hyena (Fig 5). This coincided with the time course of excreted radiolabelled fTM as the first sample collected 15.5 h following ^3^H-testosterone injection contained the highest amount of radioactivity (Fig 2). In spotted hyenas, little is known about the food transit time: it is thought to take approximately 24 h but may increase as the food volume ingested increases [50]. Dloniak *et al*., 2004 observed a delay of 24–72 h in detecting fAM in response to the injection of luteinising hormone-releasing-hormone (LHRH) [22]. In comparison, an increase in fGM concentrations was detected 16–96 h following an ACTH challenge experiment [45]. Taken together the studies imply that the time lag of faecal testosterone metabolite excretion detected by the epiandrosterone EIA corresponds to the gut passage time.

The peak in fTM levels detected 72 h pre-treatment cannot be attributed to any known event in the area around the animals’ enclosure (Fig 5) but may be associated with the movement of the animal from its normal enclosure to an enclosure at the NZG hospital during the experiment. Alternatively, androgens are known precursors of oestrogens [66] and hence in female mammals, a rise in fTM levels could be of ovarian origin. For example, elevated fTM concentrations are a reliable indicator of the follicular phase in female sun bears [60]. In female domestic dogs, fTM peaks and serum testosterone peaks are positively correlated with the day of the pre-ovulatory surge of LH [67, 68], and in female domestic pigs (*Sus scrofa*) plasma testosterone concentrations showed a significant increase 2 days before the LH peak [69]. Thus we cannot exclude the possibility that the pre-treatment fTM peak in the female spotted hyena (Fig 5) was of ovarian origin and related to oestrus.

The second step in the physiological validation of our EIA was to test our expectation that wild adult immigrant males in spotted hyena clans would show higher fTM concentrations than juvenile natal males. Our results confirmed this prediction as fTM concentrations in adult immigrant males were significantly higher than those in juvenile males (Fig 8). We currently cannot exclude the possibility that juvenile males and immigrant, adult males metabolise testosterone differently, but we are unaware of any study indicating that this is likely to be the case. Hence, we assume that the detected differences between cubs and adults indicate an increase in testosterone concentrations in reproductively active immigrant males. Furthermore, as expected we found considerable variation in fTM concentrations in both juvenile natal males and adult immigrant males. We suspect this variation reflects the differences between individuals in the frequency with which they “win” social interactions with other clan members as suggested by the “challenge hypothesis” [9]. Further studies will have to determine to what extent individual differences in fTM are related to the context and outcomes of behavioural interactions, reproductive status, social status, age or season, or possibly other behavioural or environmental factors. Our epi-A EIA provides the required tool to rigorously test these ideas.

## Supporting Information

S1 Dataset Data on faecal testosterone metabolite concentrations in spotted hyenas. (PDF)

S1 Fig Comparision of epi-A immunoreactivity to immunoreactivities of control steroids. HPLC elution fractions from the captive female were analysed in ther cortisol-21, corticosterone-21, testosterone and DHT EIAs in comparison to the epi-A EIA.
(TIF)
