# Correction: Measuring Faecal Epi-Androsterone as an Indicator of Gonadal Activity in Spotted Hyenas (*Crocuta crocuta*)

**DOI:** 10.1371/journal.pone.0163851

**Published:** 2016-09-23

**Authors:** Susanne Pribbenow, Marion L. East, Andre Ganswindt, Adrian S. W. Tordiffe, Heribert Hofer, Martin Dehnhard

Fig 7 contains an error on the x-axis. Fecal testosterone metabolites (fTM) and fecal glucocorticoid metabolites (fGM) values that are plotted on day 2 after an ACTH injection should be plotted on day 1, and the values that are plotted on day 1 should be plotted on day 2. Please see the corrected Fig 7 here.

**Fig 7:**
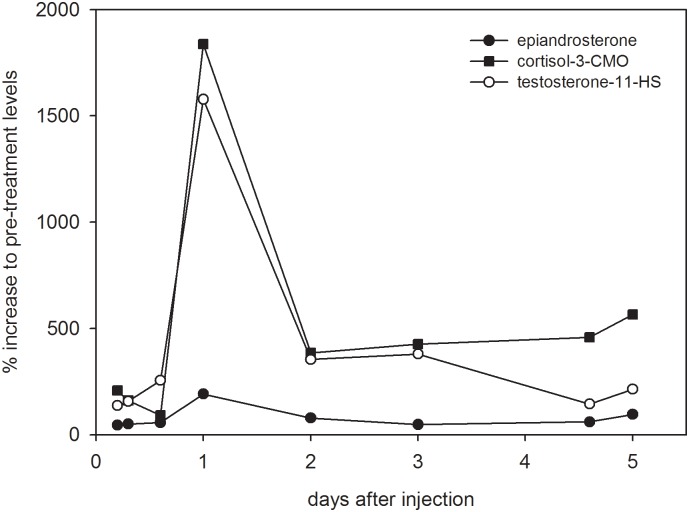
Comparison of faecal cortisol and testosterone immunoreactivity. Changes in fGM and fTM concentrations were determined in faecal samples from the ACTH challenge following hydrolysis with β-glucuronidase from *Helix pomatia* in the cortisol-3CMO, epiandrosterone and testosterone-11-HS EIAs, respectively. Levels of fGM and fTM are shown as percentage increase over pre-injection levels.

## References

[1] Pribbenow S, East ML, Ganswindt A, Tordiffe ASW, Hofer H, Dehnhard M (2015) Measuring Faecal Epi-Androsterone as an Indicator of Gonadal Activity in Spotted Hyenas (*Crocuta crocuta*). PLoS ONE 10(6): e0128706 doi: 10.1371/journal.pone.0128706 26107516 10.1371/journal.pone.0128706 PMC4481319

