# Supplementary material for: Measuring Faecal Epi-Androsterone as an Indicator of Gonadal Activity in Spotted Hyenas (Crocuta crocuta)
Source: PLoS One. 2015 Jun 24;10(6):e0128706. doi: 10.1371/journal.pone.0128706 (PMC4481319; doi:10.1371/journal.pone.0128706)
Supplement: S1 Dataset — (PDF) [file pone.0128706.s001.pdf]

Data on „Measuring faecal epi-androsterone as an indicator of gonadal activity in spotted hyenas (*Crocuta crocuta*)" S Pribbenow, ML East, A Ganswindt, ASW Tordiffe, H Hofer, M Dehnhard

1. HPLC analyses of steroid standards. Standards are measured in the respective EIA.

	testosterone	cortisol	corticosterone	dihydrotestosterone	epi-A
elution fraction	pg/20 µl	pg/20 µl	pg/20 µl	pg/20 µl	pg/20 µl
1	0,6	0,9	1,6	1,2	0,0
2	1,1	2,8	7,3	3,7	1,9
3	0,5	1,6	1,5	2,1	1,1
4	0,2	0,6	0,9	1,6	0,6
5	0,0	0,6	0,9	2,1	0,5
6	0,4	0,6	1,0	1,5	0,7
7	0,0	0,8	0,8	2,6	1,5
8	0,0	0,4	0,0	0,0	0,8
9	0,0	0,4	0,2	0,5	0,0
10	0,0	0,4	0,8	1,0	1,5
11	0,0	2,8	0,7	0,5	1,0
12	0,0	91,3	0,6	0,9	0,0
13	0,4	78,0	0,5	1,0	0,0
14	0,0	5,0	0,3	0,9	0,9
15	0,0	1,1	0,3	1,5	1,9
16	0,0	0,4	0,0	0,8	1,0
17	0,0	0,4	0,0	0,0	0,0
18	0,0	0,4	1,0	0,5	1,7
19	0,0	2,2	1,1	0,0	1,4
20	0,0	0,4	0,8	0,8	0,0
21	0,0	0,4	1,8	0,8	0,0
22	0,0	0,5	34,7	1,0	0,6
23	0,0	0,4	1215,6	1,2	1,6
24	0,0	0,4	280,1	0,7	1,0
25	0,0	0,0	12,3	0,0	0,6
26	0,6	0,4	8,3	0,0	1,8
27	0,0	0,4	2,9	0,0	1,7
28	0,0	0,4	1,6	0,7	0,0
29	0,0	0,4	1,1	0,4	0,0
30	0,0	0,4	1,1	0,0	0,4
31	0,0	0,4	2,6	1,1	1,8
32	0,0	0,3	1,2	0,0	0,5
33	0,0	0,4	1,3	1,0	0,0
34	0,0	0,4	1,5	0,9	1,8
35	100,0	3,9	0,9	1,4	1,2
36	236,3	3,4	0,9	4,5	0,0
37	14,4	0,6	0,0	0,9	0,0

	testosterone	cortisol	corticosterone	dihydrotestosterone	epi-A
elution fraction	pg/20 µl	pg/20 µl	pg/20 µl	pg/20 µl	pg/20 µl
38	2,7	0,4	0,0	0,9	0,0
39	0,7	0,4	0,0	1,3	23,9
40	0,3	0,4	0,9	186,9	78,1
41	0,3	0,4	3,6	635,4	7,0
42	0,0	0,4	1,0	244,0	2,8
43	1,5	0,4	0,4	24,6	1,5
44	0,8	0,4	0,5	6,0	0,6
45	0,0	0,4	0,0	2,0	0,5
46	0,0	0,4	6,6	1,4	1,4
47	0,0	0,4	0,0	1,2	1,7
48	0,0	0,4	0,4	1,2	1,0
49	0,0	0,4	0,2	0,9	0,9
50	0,0	0,4	0,3	1,0	2,2
51	0,0	0,4	0,0	1,0	1,0
52	0,0	0,4	0,0	1,5	0,7
53	0,0	0,4	0,0	3,0	0,8
54	0,0	0,4	0,0	1,2	0,8
55	0,0	0,6	0,0	0,9	2,1
56	0,0	0,4	0,5	1,1	1,2
57	0,0	0,4	0,5	0,8	0,6
58	0,0	0,4	0,3	1,7	2,1
59	0,3	0,4	0,0	0,8	1,1

2. Radiometabolism study female A

Lab_ID	hr after injection	radioactivity (cpm/100µl)
38	-144,0	0
40	-120,0	0
42	-96,0	0
44	-72,0	0
46	-24,0	0
48	-1,3	0
49	15,5	855
50	22,3	629
51	44,7	214
52	45,0	370
54	67,0	112
56	69,5	39
61	118,0	13
62	118,5	7
64	141,8	6
66	167,0	0

Lab_ID	hr after injection	radioactivity (cpm/100µl)
67	172,5	0
70	189,0	0

2.1. HPLC analyses of sample 49 after C18 purification

DF	epi-A (pg/20µl)	epi-A (pg/200µl)	cpm/200µl
1000	1,19	11930,0	7600

**before hydrolysis**			**after hydrolysis**					
		epi-A EIA		epi-A EIA	cortisol-21 EIA	Cortico-sterone-21 EIA	testosterone-EIA	DHT-EIA
elution fraction	cpm/100µl	pg/20µl	cpm/100µl	pg/20µl	pg/20µl	pg/20µl	pg/20µl	pg/20µl
1	1	1,3	6	0,9	4,9	3,4	3,9	5,9
2	962	185,1	6	36,0	5,3	10,1	2,7	5,9
3	478	805,0	8	6,6	4,4	7,5	2,0	3,4
4	164	922,3	6	1,2	3,3	7,0	2,3	4,5
5	36	102,3	18	1,1	1,8	8,0	1,4	4,2
6	24	34,4	0	1,1	3,8	7,7	1,7	4,6
7	7	17,8	11	1,8	0,6	2,7	0,9	1,4
8	8	24,3	2	1,6	1,3	4,5	1,6	2,3
9	10	20,6	7	2,8	0,5	0,7	0,7	1,4
10	5	16,6	7	2,4	0,5	0,8	0,8	1,5
11	3	16,9	3	2,3	4,5	10,6	2,9	5,2
12	8	12,1	12	1,7	0,3	0,7	0,5	0,9
13	6	6,7	0	1,8	0,4	1,2	0,7	1,4
14	16	8,8	4	1,5	0,2	1,2	0,8	1,2
15	14	9,5	5	4,0	0,6	1,4	0,9	9,0
16	14	6,7	4	2,6	0,7	1,3	3,9	3,3
17	11	7,2	14	3,4	0,4	0,3	2,0	1,3
18	3	5,8	16	4,5	0,5	0,8	1,0	1,1
19	10	3,8	4	3,4	0,7	1,0	0,8	1,2
20	5	3,8	13	4,4	9,8	13,1	5,3	5,4
21	2	3,0	2	2,7	0,4	1,4	1,7	1,7
22	9	8,8	7	5,3	0,3	1,4	2,3	1,6
23	10	6,1	11	23,5	7,2	13,4	4,6	6,3
24	6	6,6	10	51,0	2,2	3,2	2,4	2,5
25	9	21,3	2	42,6	2,5	5,9	5,9	4,0
26	45	44,9	16	49,0	10,6	12,2	12,4	7,6
27	10	15,0	18	13,3	2,0	2,5	2,8	2,4
28	12	17,7	11	15,0	1,9	5,9	10,1	6,0
29	23	35,4	8	23,0	7,3	12,3	6,1	7,0
30	23	14,1	10	10,3	3,3	4,8	2,3	2,5
31	13	16,0	17	10,6	1,7	3,7	1,7	2,0

before hydrolysis			after hydrolysis					
		epi-A EIA		epi-A EIA	cortisol-21 EIA	Cortico-sterone-21 EIA	testosterone-EIA	DHT-EIA
elution fraction	cpm/100µl	pg/20µl	cpm/100µl	pg/20µl	pg/20µl	pg/20µl	pg/20µl	pg/20µl
32	12	8,0	4	8,0	1,6	3,8	0,9	1,3
33	10	6,4	8	7,9	2,9	3,2	1,5	7,3
34	10	13,7	6	15,7	11,4	7,2	1,2	1,6
35	16	11,0	1	10,9	26,6	24,2	3,0	4,9
36	9	18,9	4	17,5	9,1	22,1	2,0	2,9
37	5	9,7	0	12,0	1,6	6,9	1,0	3,7
38	3	43,3	6	33,4	9,1	18,5	1,9	6,5
39	11	48,8	27	42,2	35,4	28,5	2,9	12,7
40	26	194,3	54	282,8	28,9	17,3	9,8	20,0
41	5	40,5	26	42,7	21,1	11,4	5,1	11,5
42	13	16,2	4	12,4	10,5	6,8	1,4	2,7
43	10	13,2	14	5,4	9,3	8,2	1,3	1,6
44	7	11,8	13	2,8	6,2	10,7	0,9	1,5
45	5	10,3	12	5,2	2,3	2,8	0,9	4,7
46	11	9,4	0	4,8	9,7	21,9	1,8	2,8
47	11	5,9	10	6,0	6,1	23,7	1,6	3,7
48	8	7,2	15	5,6	16,4	23,3	1,5	3,5
49	3	4,3	10	3,8	7,1	8,9	1,2	2,9
50	5	7,1	8	9,0	26,8	37,0	4,9	9,6
51	5	9,0	13	6,1	2,9	7,0	1,5	3,8
52	9	8,5	6	7,8	2,1	6,2	0,9	24,6
53	6	3,6	4	4,1	1,7	8,5	0,8	2,4
54	0	5,0	4	2,4	1,2	5,2	0,5	2,6
55	0	7,1	4	5,9	0,8	3,3	1,1	1,6
56	0	8,4	12	3,5	0,8	3,1	1,2	1,8
57	7	6,0	5	2,4	2,9	2,8	1,9	5,9
58	7	4,2	5	2,7	0,0	1,7	0,9	1,5
59	0	5,8	5	3,4	0,0	2,0	0,0	1,3

3. Radiometabolism study male A

Lab_ID	hr after injection	radioactivity (cpm/100µl)
5	-18,2	37
9	-1,5	29
12	6,9	37
32	47,3	1041
36	71,3	263

3.1. HPLC analyses of sample 32 after C18 purification

DF	epi-A (pg/20µl)	epiA (pg/50µl)	cpm/50µl
1000	1,95	4875,0	2250

**before hydrolysis**			**after hydrolysis**	
		epi-A EIA		epi-A EIA
elution fraction	cpm/100µl	pg/20µl	cpm/100µl	pg/20µl
1	94	1,4	14	0,0
2	74	3,0	7	2,2
3	133	3,8	7	2,0
4	13	4,1	17	3,8
5	11	2,8	6	1,8
6	8	2,8	4	1,5
7	4	3,8	16	1,3
8	10	3,8	9	2,2
9	7	2,3	4	1,8
10	9	3,1	9	1,9
11	3	4,2	10	1,8
12	15	3,2	9	5,9
13	14	2,9	14	1,5
14	18	4,2	8	1,7
15	10	5,5	7	2,4
16	4	7,5	13	5,5
17	5	3,6	16	3,9
18	0	3,5	10	2,7
19	9	4,9	8	5,4
20	8	6,6	16	10,8
21	5	22,4	12	5,5
22	9	26,3	8	24,8
23	11	13,1	3	6,9
24	9	14,9	10	5,8
25	13	15,1	25	16,2
26	7	5,2	11	20,1
27	8	7,5	9	16,1
28	4	16,3	6	10,6
29	3	24,5	13	5,2
30	6	22,5	6	13,3
31	8	30,1	25	9,9
32	6	20,2	15	7,9
33	8	14,0	7	17,6
34	3	21,9	23	18,7
35	15	16,7	13	15,0
36	13	22,3	18	19,7
37	19	20,1	18	15,6

before hydrolysis			after hydrolysis	
		epi-A EIA		epi-A EIA
elution fraction	cpm/100µl	pg/20µl	cpm/100µl	pg/20µl
38	8	8,4	22	26,3
39	8	19,0	13	25,9
40	30	7,5	74	92,5
41	19	5,1	21	19,8
42	19	14,8	23	8,7
43	18	6,9	7	4,5
44	16	7,1	16	3,0
45	19	3,9	40	7,3
46	20	2,9	17	9,4
47	9	7,1	18	4,6
48	12	1,8	17	20,7
49	5	2,0	13	1,3
50	1	3,0	11	7,3
51	6	2,0	9	11,8
52	14	2,5	10	4,0
53	8	2,8	7	2,2
54	7	2,5	17	2,3
55	6	1,7	17	3,0
56	14	0,9	3	10,0
57	5	0,0	1	2,1
58	13	1,1	5	1,7
59	9	4,3	12	2,0

4. HPLC analyses of faecal samples from free-ranging hyenas

4.1. Female B after C18 purification

DF	epi-A (pg/20µl)	epiA (pg/150µl)
10	31,26	2346,6

before hydrolysis		after hydrolysis
	epi-A EIA	epi-A EIA
elution fraction	pg/20µl	pg/20µl
1	2,4	1,6
2	4,8	2,0
3	8,9	1,0
4	3,9	1,5
5	2,5	2,5
6	2,6	1,2
7	3,4	2,2
8	4,3	3,0

**before hydrolysis**		after hydrolysis
	epi-A EIA	epi-A EIA
elution fraction	pg/20µl	pg/20µl
9	10,1	2,8
10	2,4	3,1
11	1,6	3,9
12	4,6	3,5
13	2,1	6,2
14	2,8	3,2
15	3,4	4,2
16	3,1	2,9
17	4,5	2,1
18	4,3	3,6
19	6,1	3,8
20	9,3	4,8
21	2,5	4,7
22	6,5	4,6
23	6,5	4,4
24	8,6	4,1
25	20,4	17,4
26	26,2	18,2
27	11,2	5,7
28	7,6	5,2
29	8,2	5,9
30	2,8	4,4
31	5,2	4,1
32	4,9	2,5
33	11,7	5,9
34	9,2	5,5
35	11,1	5,4
36	10,3	4,8
37	14,6	9,3
38	62,1	37,4
39	36,8	24,5
40	176,2	89,9
41	23,8	17,3
42	9,6	6,1
43	6,3	5,0
44	3,8	4,8
45	45,2	3,6
46	17,5	15,5
47	13,8	9,8
48	20,9	12,7
49	13,4	8,6
50	20,2	18,8
51	14,5	7,0

**before hydrolysis**		after hydrolysis
	epi-A EIA	epi-A EIA
elution fraction	pg/20µl	pg/20µl
52	5,9	4,4
53	4,2	2,8
54	3,1	4,0
55	5,4	4,3
56	6,0	5,8
57	3,7	6,1
58	5,8	5,8
59	3,1	5,0

4.2. Male B after C18 purification

DF	epi-A (pg/20µl)	epiA (pg/150µl)
10	51,31	3852,5

**before hydrolysis**		after hydrolysis
	epi-A EIA	epi-A EIA
elution fraction	pg/20µl	pg/20µl
1	3,9	0,0
2	4,8	0,0
3	5,6	1,4
4	4,1	0,0
5	12,5	0,0
6	2,6	0,0
7	2,7	0,0
8	2,9	1,1
9	2,5	0,0
10	4,4	0,0
11	2,0	3,2
12	4,8	0,6
13	4,9	0,0
14	4,1	0,0
15	5,7	0,0
16	4,2	3,9
17	4,4	0,0
18	5,3	0,0
19	4,9	2,0
20	6,9	2,1
21	4,5	0,0
22	6,5	0,0
23	7,6	1,7
24	9,3	5,1
25	30,7	2,9

**before hydrolysis**		after hydrolysis
	epi-A EIA	epi-A EIA
elution fraction	pg/20µl	pg/20µl
26	31,6	0,7
27	8,8	3,2
28	10,3	0,6
29	16,7	0,0
30	9,0	0,0
31	8,7	2,0
32	6,2	2,6
33	9,1	0,0
34	8,4	0,0
35	7,7	0,0
36	10,4	0,0
37	10,6	2,6
38	47,1	3,1
39	35,9	5,7
40	200,0	35,4
41	69,5	5,1
42	13,1	3,5
43	10,6	12,6
44	7,5	1,0
45	6,9	0,4
46	7,5	0,0
47	6,8	0,0
48	6,9	2,2
49	8,4	0,0
50	7,1	1,9
51	6,5	0,9
52	6,4	1,9
53	4,1	3,6
54	3,4	0,0
55	9,0	1,6
56	4,3	2,9
57	7,2	0,0
58	7,0	0,6
59	4,9	2,6

5. Testosterone challenge

Lab_ID	hr after injection	epi-A (µg/g)
38	-144,00	0,6
40	-120,00	0,1
42	-96,00	0,8
44	-72,00	3,7

Lab_ID	hr after injection	epi-A (µg/g)
46	-24,00	0,7
48	-1,25	0,7
49	15,50	3,2
50	22,25	0,6
51	44,66	0,7
52	45,00	1,0
54	67,00	0,6
56	69,50	0,9
61	118,00	0,7
62	118,50	0,7
64	141,75	1,0
66	167,00	1,0
67	172,50	2,1
70	189,00	1,3

6. Comparison of *Hp* and *Ec* glucuronidases

hr after injection	*Hp* (µg/g)	*Ec* (µg/g)
-144,0	0,6	0,2
-120,0	0,9	0,7
-96,0	1,0	0,9
-72,0	2,1	1,3
-24,0	0,9	0,4
-1,3	0,5	0,2
15,5	1,3	0,8
22,3	0,6	0,3
44,7	0,2	0,3
45,0	0,5	0,5
67,0	0,5	0,3
69,5	0,2	0,2
118,0	0,2	0,0
118,5	0,3	0,2
141,8	0,3	0,2
167,0	0,5	0,4
172,5	0,3	0,4
189,0	0,6	0,3

7. Comparison of fTM from juvenile males and adult immigrant males

Sex	epi-A (ng/g)
juvenile1	391,9
juvenile2	800,5
juvenile 3	975,2
juvenile 4	555,9

Sex	epi-A (ng/g)
juvenile 5	750,9
juvenile 6	1126,6
juvenile 7	340,5
juvenile 8	1277,8
juvenile 9	423,3
juvenile 10	272,7
juvenile 11	1295,1
juvenile 12	1232,7
juvenile 13	647,4
juvenile 14	513,2
juvenile 15	384,1
male1	1184,8
male2	2173,3
male3	2451,9
male4	2434,9
male5	1877,9
male6	1255,1
male7	4259,8
male8	804,2
male9	355,1
male10	179,3
male11	2310,5
male12	1767,3
male13	716,2
male14	283,9
male15	1566,0

- juvenile males

mean: 732,5 ng/g

SD: 365,2 ng/g

- adult immigrant males

mean: 1574,7 ng/g

SD: 1083,4 ng/g
