# Supplementary figures and images for: Measuring Faecal Epi-Androsterone as an Indicator of Gonadal Activity in Spotted Hyenas (Crocuta crocuta)
Source: PLoS One. 2015 Jun 24;10(6):e0128706. doi: 10.1371/journal.pone.0128706 (PMC4481319; doi:10.1371/journal.pone.0128706)

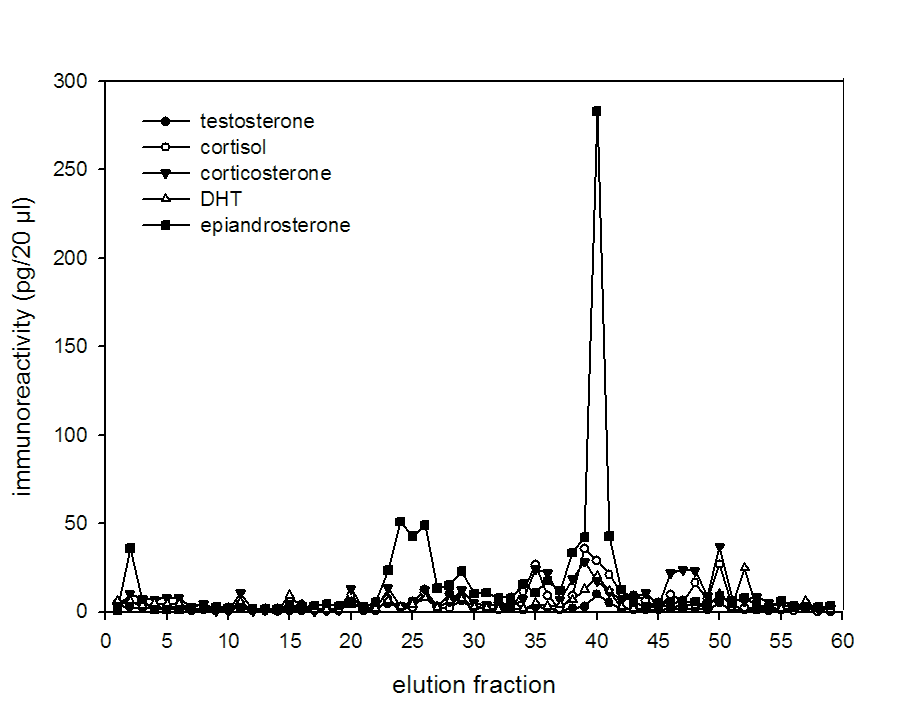

Supplement: S1 Fig — HPLC elution fractions from the captive female were analysed in ther cortisol-21, corticosterone-21, testosterone and DHT EIAs in comparison to the epi-A EIA. (TIF) [file pone.0128706.s002.TIF]
